# The Puncture and Water Resistance of Polyurethane- Impregnated Fabrics after UV Weathering

**DOI:** 10.3390/polym12010015

**Published:** 2019-12-19

**Authors:** Antonella Patti, Domenico Acierno

**Affiliations:** 1 Department of Civil Engineering and Architecture, University of Catania, Viale Andrea Doria 6, 95125 Catania, Italy; antonella.patti@unict.it; 2 CRdC Nuove Tecnologie per le Attività Produttive Scarl, Via Nuova Agnano 11, 80125 Naples, Italy

**Keywords:** waterborne polyurethane dispersion, UV light stabilizers, impregnation, puncture resistance, water repellency

## Abstract

Polyurethane is a polymer adaptable to different scientific and industrial requirements; nevertheless it is also extremely susceptible to UV radiation, which compromises the physical and mechanical functionality. In this framework, our study investigated the effect of waterborne polyurethane dispersion (WPUD) applied to a polyester (PET)-based fabric, through the impregnation method, on the puncturing and water resistance of the pristine material, before and after UV weathering. Results confirmed an increment of both features in the prepared fabrics, attributed to the PUR textile treatment; but a partially loss of the gained properties in the samples due to the UV weathering. In order to improve the efficiency of the impregnating dispersions, in protecting the durability of the treated materials, the addition of different UV light stabilizers, or/and of crosslinking agent into WPUD was also tested. From the experimental data, it can be concluded that formulations based on WPUD, containing both the crosslinker and UV organic absorber, have displayed an increment of their perforation and water resistance for the treated samples with respect to the starting textile, and contemporary have preserved the features against the UV light. Finally, microscopic and spectroscopic analyses have been performed as further characterization techniques of the samples surface.

## 1. Introduction

Polyurethanes (PURs) are a class of polymers (thermoplastic or thermosetting) obtained by a chemical reaction between aliphatic or aromatic isocyanate groups (R-(N=C=O)_n_) with polyols (R′-(OH)_n_), mainly polyester or polyether-based. While the first reagents constitute the hard segments and provide the strength and the rigidity of the PUR based-materials, the second constituents represent the soft segments and provide the elasticity and flexibility features. Thus, PURs have attracted great scientific and industrial attention, due to the versatility of the pristine constituents [1,2], that provide this polymer a behavior adaptable for foams [3], composite materials [4], coatings and adhesives [5]. Particularly, PURs are the mostly used polymers in the textile coating industry because they may be made strong and rigid or soft and elastic by varying their chemical structures. They possesses good washproofness and cleaning resistance, good adhesion to fabrics, good chemical and abrasion resistance, along with a pleasurable and soft to the touch surface. They can be applied to textiles and leather, in different forms such as granules or powders, solutions or water-based dispersions, depending on the manufacturing technologies [6]. However, the production of traditional PUR polymers is based on petroleum, as the main raw material, and this implies the adverse emission of volatile organic compounds (VOC) and other harmful substances. Therefore, due to these environmental pollution and petroleum depletion concerns, scientific research attention has been paid to study eco-friendly alternatives, i.e., waterborne polyurethane dispersions (WPUDs), with very low VOC emissions and with water as a low-cost, safe for health and the environment, and non-toxic solvent [7]. The aqueous polyurethane characteristics derive essentially from the chemical structure of urethane groups, by which multiple hydrogen bonds between adjacent polymer chains can take place. The chemical arrangement is constituted through hard segments that align in a dry polymer, and self-assemble into strongly hydrogen-bonded areas via flexible soft segments. The multiplicity of strong hydrogen bonds provides the properties commonly associated with a covalently bonded network [8].

In the literature, different works have explored the possibility of developing green methods for the synthesis of WPUDs which can be applied as coatings, adhesives and finishing agents, useful for industrial production and application. The results have confirmed that WPUD coatings are endowed with excellent thermal stability, waterproofness and good mechanical properties, in terms of elongation at break and tensile strength [9,10,11,12].

In general, the exposure of a polymer to UV radiation causes breakage of the chemical bonds in the material, a subsequent reduction of its molecular weight and a loss in its mechanical features (“photo-oxidative degradation”) [13]. Since polyurethane coatings are also utilized for outdoor applications, high durability and weathering resistance may be required for the ambient exposure to ultraviolet (UV) radiation, water/humidity, and temperature. Among the different strategies to overcome the photo-oxidation of PUR-based materials, the addition of organic UV absorbers, hindered amine light stabilizers (Hals) or functional particles (TiO_2_, ZnO) into the coating formulations has been studied [14]. The action of these additives against the UV weathering is different: in details, the inorganic particles behave by scattering the light; on the contrary, the organic UV absorbers convert UV light into heat and higher wavelengths, while radical interceptors (Hals) capture the free radicals of the starting polymeric decomposition and prevent to this process from continuing. The organic UV absorbers seem to be more efficient compared than the inorganic particles yet their durability is poor due to evaporation and migration from the surface [15].

In the attempt to increase the waterproofness and the puncture resistance of a commercial technical polyester-based (PET) woven fabric for possible use in semi-rigid luggage production, this work studied the application of waterborne polyurethane dispersions to the fabric surface through the impregnation method. This technique was preferred to coating—the most common approach—in order to act as reinforcement and protection of the fabric weaving without covering the surface and altering the textile appearance. The features of interest were also analyzed after UV-accelerated aging, verifying the durability of the developed products over time. Then, an optimal formulation of the impregnating PUR dispersions has been also investigated by the addition of the UV light stabilizers (such as organic UV absorbers, hindered ammine light stabilizer and zinc oxide nanoparticles) or/and crosslinker, in order to improve the endurance and the duration of the final materials.

## 2. Materials and Methods

### 2.1. Materials

A commercial polyester-based woven fabric (100% PET, areal mass = 300 g/m^2^) was supplied by FLUKSO, Udine, Italy. The waterborne dispersion of polyester-based aliphatic polyurethane (IMPRANIL DL 1380, 58 wt % of solid content) was kindly furnished by Covestro, Leverkusen, Germany. An aliphatic polyisocyanate-based crosslinking agent (Cr), identified as ICAPLINK X3, was kindly supplied by ICAP-Sira Chemicals and Polymers spa, Parabiago-Milan, Italy. Zinc oxide (ZnO, average particle size of 100 nm), was purchased from Sigma Aldrich Co. LLC., Milan, Italy. An aqueous dispersion of an oligomeric hindered amine light stabilizer (HALS), named HOSTAVIN 3070 DISP (active component content of 52 wt %), and an aqueous dispersion of a 2-hydroxy-phenyl-s-triazine UV absorber (HPT), named Tinuvin 400-DW (N) (active component content of 20 wt %), specifically designed for UV stabilization of waterborne coatings, were provided by Clariant, Konstantynów Łódzki, Poland, and BASF Italia spa, Cesano Maderno, Italy, respectively.

### 2.2. Sample Preparation

A square piece of fabric (20 × 20 cm^2^) was impregnated with the commercial WPUD (PUD/Fab), and dried in climatic chamber (mod. 250E, Angelantoni Industrie spa, Perugia, Italy) at controlled temperature (25 °C) and humidity (50%). When the additives were introduced into impregnating solutions, all the constituents were mixed under magnetic stirring at 800 rpm for 15 min (T = 25 ± 2 °C and RH = 50% ± 4%). Formulations, containing 10% in wt. of crosslinker (PUD/10Cr), and/or up to 7% in wt. of active components of UV light stabilizers, were prepared in the compositions reported in Table 1. The percentages of the ingredients, referred to the nominal PUR solid content into the IMPRANIL dispersion (~7.8 g), were set in agreement with the manufacturer suggestion on the technical datasheet. For PUD/XA and PUD/10Cr/XA, it was intended with X the weight content of the additive and A the typology of UV stabilizer (ZnO, Hals or HPT).

After the impregnation the prepared samples were immediately weighed in order to make sure that the amount of dispersion applied (~13 g) was always the same. Then, the measurement of the weight samples was continued for evaluate the time when all the water was evaporated and the drying process was terminated. The tests were carried out on treated specimens after 4 days in accordance with the drying time and low temperature cure reaction.

**Figure 1:**
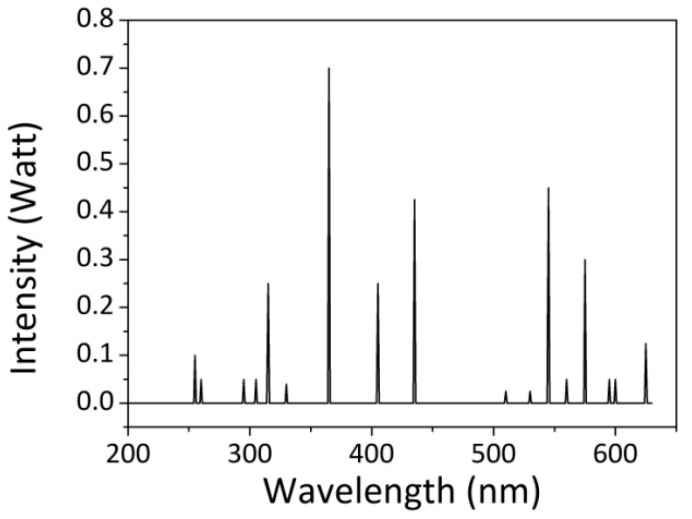
UV lamp emission spectrum.

**Figure 2:**
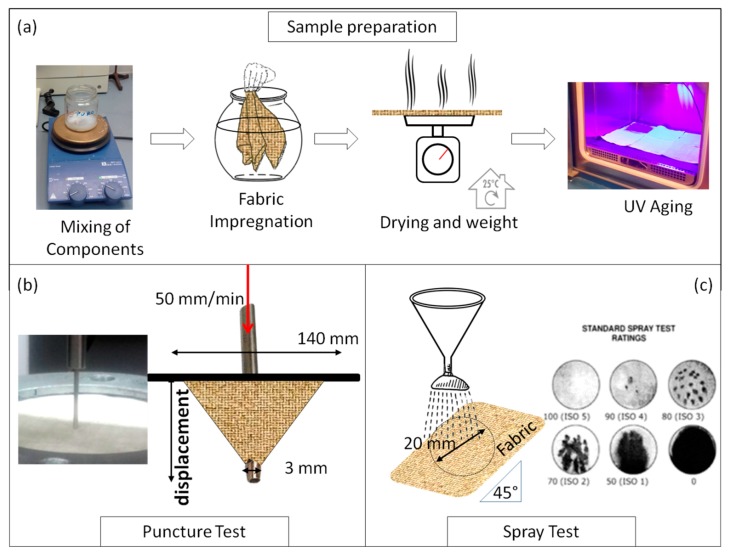
Schematic representation of (**a**) sample preparation; (**b**) puncture test method; (**c**) spray test equipment.

When required, the UV accelerated aging was carried out in the environmental chamber equipped with a UV bulb lamp (wavelength range of 300–600 nm, shown in Figure 1) in dry air at 35 °C for 8 days. The preparation method of the impregnated and aged fabric samples is reported in Figure 2a.

### 2.3. Characterization Techniques

The puncture strength of the developed specimens was measured on a dynamometer TENSOMETER 2020 by Alpha Technologies INSTRON (Norwood, MA, USA), equipping the upper mobile grip with a rounded tip spike (3 mm in diameter). The square sample, 20 × 20 cm^2^ in size, was placed between two annular flanges with an internal diameter of 14 cm, carefully fixed in turn with four screws by preventing sample slippage.(as shown in Figure 2b). The crosshead was moved downward, perpendicularly to the sample position, at a running speed of 50 mm/min. The highest point on the load displacement curve, corresponding to the maximum load recorded at the sample breaking (by using the software Tensile 2020), was considered the puncture resistance of the tested fabric. All tests were repeated at least on 10 samples.

The water repellency was determined by a spray test (STA Branca Idealair, Varese, Italy, according to the standard UNI EN 24920, as seen in Figure 2c). For the purpose, 250 mL of distilled water was poured, by a suitable dispenser on the surface of fabric mounted on a circular frame inclined at 45°. The wettability index (ISO) was determined by comparing the appearance of tested surface with a photographic scale of five possible values ranging from ISO 0 to ISO 5, corresponded to totally wettable and completely water repellent surface, respectively.

The polyurethane distribution on the textile yarns was investigated through an emission scanning electron microscope (SEM, Mod. TM 3000, by Hitachi Company, Tokyo, Japan). High vacuum conditions have been used and tests were performed on the fabric surface after the gold metallization.

The alteration in fabric aesthetics due to the different treatments was attested by using an optical microscope (Olympus SZ-PT, Tokyo, Japan) equipped with a digital camera (Olympus U-PMTVS).

The spectroscopic analysis was performed on the developed samples by using NICOLET 6700 FT-IR spectrophotometer, produced by Thermo Scientific (Waltham, MA, USA), by operating in attenuated total reflection modality (ATR) with a zinc selenide crystal, a wavenumber range of 400–4000 cm^−1^, a resolution of 4 cm^−1^ and 16 scans. For each obtained spectrum, the data have been reworked by performing baseline correction and advanced ATR correction, related to the adopted ZnSe crystal, by the OMNIC software.

The loss in perforating strength and in IR absorbance was recorded by comparing the measured quantities before and after UV aging, according to the following formula:

$$loss\;\left(\%\right)=\frac{Value_{beforeUV}-Value_{afterUV}}{{Value}_{beforeUV}}*100$$

where *V_beforeUV_* indicated the puncture strength (or IR absorbance) measured in the samples before the *UV* weathering and *V_afterUV_* indicated the corresponding value for the aged samples.

## 3. Results

### 3.1. Puncture Strength

The puncture behavior of the overall impregnated PET fabrics, before and after UV accelerated aging, is reported in Table 2 in terms of the maximum load and displacement recorded during each test. In particular the effect of the studied soaking dispersions, with different components and concentrations, on the mechanical features of the pristine PET material was highlighted as follows: in Table 2 point (a) the influence of the basic polyurethane solution, and point (b) the introduction in it of the crosslinker; in Table 2 point (c), (d) and (e) the influence of the addition in the commercial WPUD of the ZnO, Hals, or HPT; in Table 2 point (e) the influence of combining crossinker and the stabilizers into the aqueous solutions.

From the data, by comparing PET and PUD/PET samples (Table 2(a)), it can be attested that the polyurethane impregnation determined an increase of puncture strength equal to +27.5% and a quite small reduction in the displacement (~−10%). Yet, for the PUD/PET fabric, by comparing the data before and after aging, it can be observed that, following the UV weathering, these benefits completely vanished, resulting into a loss of perforation characteristics equal to −48.5% as concerning the breaking load, and equal to −15% for the displacement. On the other hand, the mechanical leak recorded for the basic PET material, due to the irradiation, resulted −13.7% in terms of maximum puncture strength, and f −34.5% regarding the displacement. In fact, as expected, the photo-oxidation produced an embrittlement of the polymer surface, which is the major cause of the damage and fracture in the polymer, by negatively and drastically affecting the mechanical features such as tensile strength, elongation, and impact strength. The brittle surface formed cracks at low strains that can propagate within the unaffected material [16]. Thus, it can be concluded that the UV treatment had completely deteriorated the positive effect arise from the polyurethane application to the fabric weaving, even bringing to a puncture strength of PUD/PET (105 ± 10 N) lower compared to that measured for the aged PET (138 ± 15 N). This result could be not only attributed to a chemical deterioration of chemical bonds within the polyurethane backbones [17] but also to a damage of the interactions between polymer and fibers [18].

When the crosslinking agent was added to the polyurethane (Table 2(b)), the load characteristics of the corresponding textiles (PUD/10Cr/PET) compared to the PUD/PET did not change significantly even if an evident reduction in the displacement (−27%) was recorded. Thus, the presence of the crosslinker caused a stiffening of the polyurethane and consequently of the whole structure. After UV aging, also for these samples, the mechanical features (strength of 115 ± 14 N) continued to be inferior compared to the starting aged PET.

As concerning the addition of the UV light stabilizer into WPUD, in the case of ZnO (Table 2(c)), no alteration of the puncture load in the respective treated fabrics was determined for an added amount equal to 4% and 7% of nanoparticles: the measured strength before UV was comparable to that of PUD/PET. On the contrary, the addition of the HALs (Table 2(d)) or of the HPT (Table 2(e)) seemed to slightly reduce the puncturing load of the corresponding systems, with respect to the PUD/PET specimens. Probably, the presence of these components had decreased the compatibility between the polyester-based fabric (PET) and the chosen commercial polyester-based polyurethane dispersion (WPUD), following a reduced adhesion between the adopted polymer and fibers. However, only in the case of the organic absorber (HPT), at the end of the UV treatment, the puncture resistance could be considered similar to that of the pristine materials (PET).

The presence of UV light stabilizers was revealed to be efficient when combined with the crosslinker agent into the WPUD Table 2(f)). In fact, before the weathering, the mechanical performances of the respective treated textiles were similar to that of PUD/PET samples and also; the damage in mechanical features due to the irradiation seemed to be less drastic. In particular, for the combination of HPT and the crosslinking agent, the breaking load of the aged samples became almost superior to that of the PET fabric.

**Figure 3:**
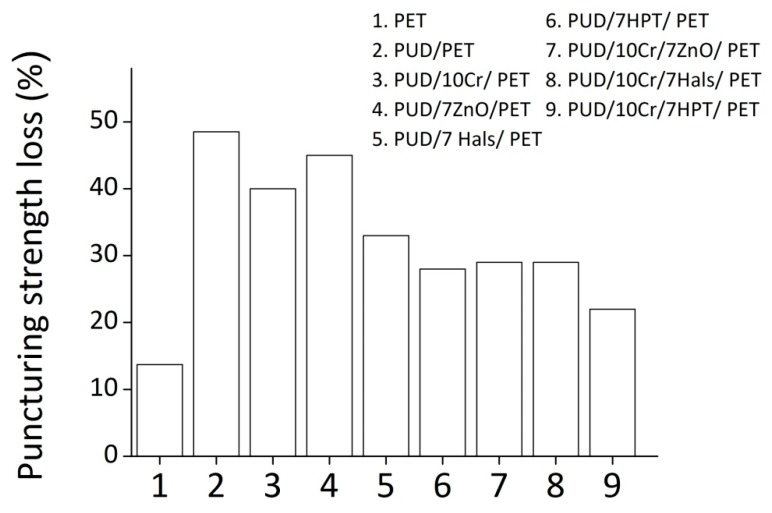
Puncture strength loss, following the UV aging, recorded for the selected samples.

In Figure 3, the loss in the puncturing strength caused by the ultraviolet rays is shown for selected samples. In details, for PUD/PET samples, the highest decrement (−48.5%) of the mechanical features has been verified; then, by adding the crosslinker (PUD/10Cr/PET), this reduction achieved the value of 40%. Furthermore, in the presence of UV light stabilizers, the reduction of the mechanical properties became equal to 28% (in the case of the HPT), and reach 22% when a combination of UV organic absorber and crosslinker was introduced into the WPUD.

### 3.2. Water Resistance

**Figure 4:**
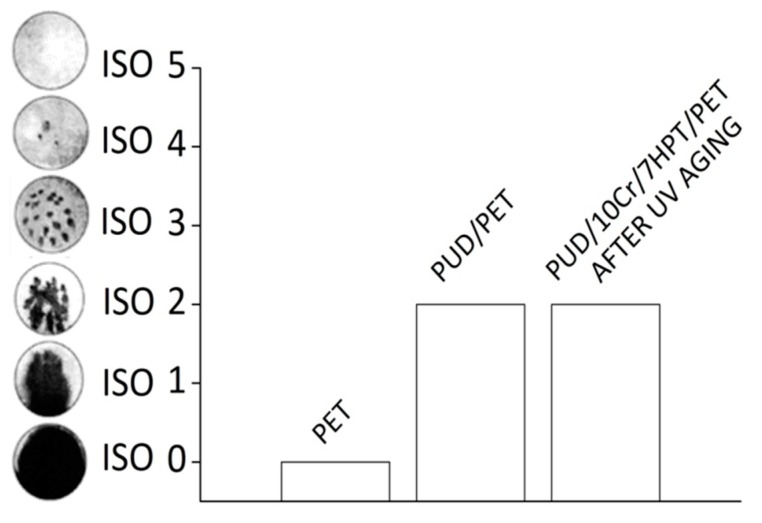
ISO INDEX for selected textiles.

The resistance of fabric to wetting has been measured by a standard spray test method. The experimental results, shown in Figure 4, demonstrated the efficiency of the polyurethane applied to the fabric weaving in improving the water resistance of the neat textile: the estimated ISO index for PUD/PET samples were about two times higher compared to the pristine PET. In fact, during the test, the starting material completely absorbed the water falling on its surface and, at the end, it appeared entirely wetted; at the same time, in the case of impregnated specimens (PUD/PET), the water drops tended to slip on the surface, being only partially absorbed by the sample. In this way, the textile face, exposed to the spraying resulted moderately sprinkled. Consequently it can be supposed that the application of the polyurethane to the textile weaving formed a protective thin and clear layer for the fibers, and contributed in filling the gaps among the filaments by limiting the passage of water. These considerations have been confirmed by SEM micrographs (shown later). Moreover, after UV aging, the impregnating dispersion, containing both the crosslinker and UV organic absorber, continued to maintain the same ability in preserving the PET fabric by the water penetration, showing the same ISO index (2), evaluated in the case of PUD/PET samples.

### 3.3. Microscopic Aspects

**Figure 5:**
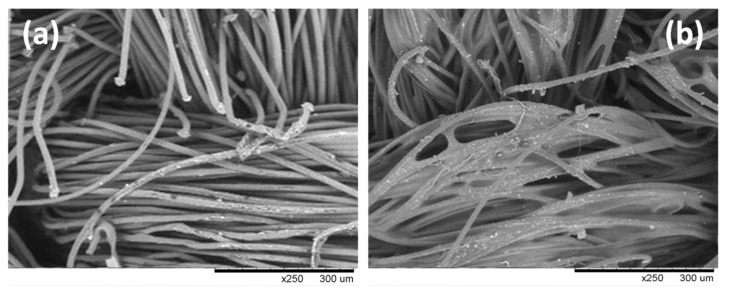

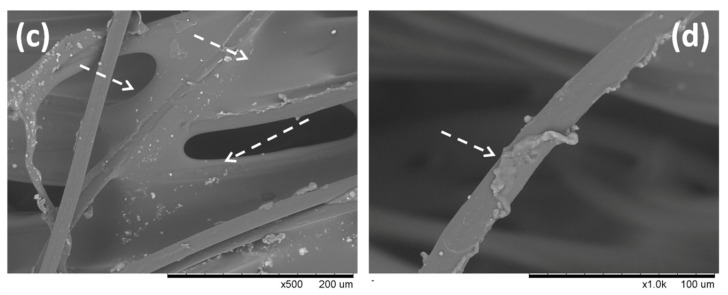
Comparison of SEM micrographs of: neat PET textile (**a**) and PUD/PET (**b**), at the same magnification (×250). SEM micrographs of PUD/PET surface at a higher magnification of ×500 (**c**) and of ×1000 (**d**). Dashed arrows are used in the figures to highlight the presence of polyurethane within the fabric weaving.

Figure 5 reports the SEM images of the two types of textile surfaces related to the neat PET textile (a) and PUD/PET impregnated samples (b). The images were obtained with same magnification (×250) for comparison. Higher magnifications of PUD/PET surface, at ×500 (c) and at ×1000 (d), respectively, have been reported for giving a more suitable visibility of the described areas. As observed in Figure 5a, for the original material, no linkage among the threads could be detectable; on the contrary, for the impregnated specimens the presence of the polyurethane, highlighted in the images with dashed arrows, was detectable among the interstices of the yarns (Figure 5b,c) and over the external part of the filaments (Figure 5d), and seemed to be responsible of the bonding and gluing points among the constituent fibers of the textile.

**Figure 6:**
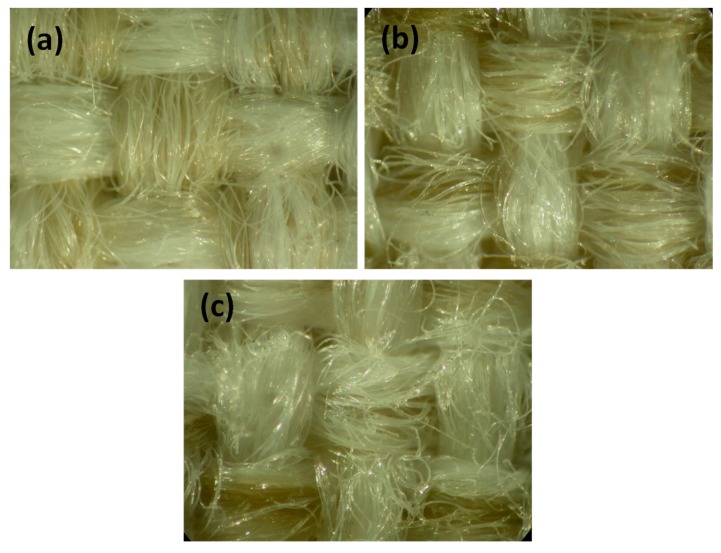
Optical microscopic images of: neat PET (**a**), PUD/PET (**b**), PUD/10Cr/7HPT/PET after UV exposure (**c**).

The effect of polyurethane treatment and UV aging on the aesthetics of the developed systems was attested by optical microscopic images, reported in Figure 6. In detail, Figure 6a displayed the outward aspects of the untreated fabric, while Figure 6b represented the surface of the PUD treated textile, and Figure 6c described the final appearance after the UV weathering of the PUD/10Cr/7HPT/PET specimen. From these pictures, no substantial changes of the surface aspect due to the different treatments could be verified. These evidences confirmed that the impregnation could be a useful treatment method for realizing a clear and transparent protection of the substrate without compromising the final visual characteristics. Moreover, in the case of the optimal studied formulation of polyurethane, crosslinker, and organic absorber, the ultimate product over time (after UV weathering) seemed to however uphold the primary aspect.

### 3.4. Spectroscopic Analysis

**Figure 7:**
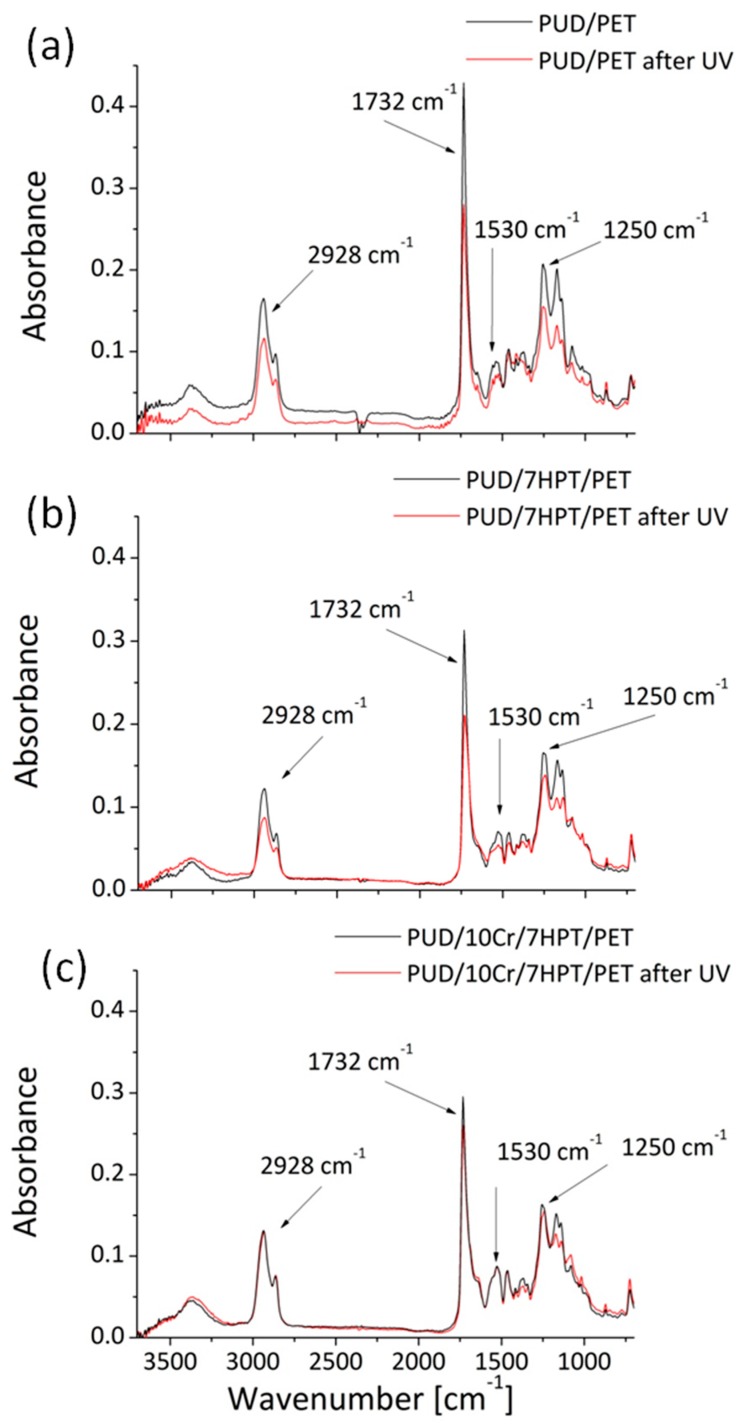
Comparison between ATR spectra of treated samples before and after UV rays: PUD/PET (**a**), PUD/7HPT/PET (**b**), and PUD/10Cr/7HPT/PET (**c**).

FT-IR spectroscopic measurements were used to identify and give a qualitative analysis of the chemical changes, occurred for the ultraviolet irradiation, in the polyurethane based-systems [17]. The ATR spectra of the studied materials, in terms of the recorded absorbance as a function of the wavenumber range in cm^−1^, are presented in the Figure 7 (PUD/PET (a), PUD/7HPT/PET (b), and PUD/10Cr/7HPT/PET (c)) by comparing for each sample the aged (red curves) and not aged (black curved) conditions.

The attention was focused on the typical adsorptions bands of the PUR chemical degradation [19]: (i) at 2928 cm^−1^ linked to asymmetric CH_2_ stretching; (ii) at 1732 cm^−1^ attributed to C=O stretching in ester and urethane groups; (iii) at 1530 cm^−1^ associated to N-H bending and C-N stretching in urethane linkages; (iv) at 1250 cm^−1^ assigned to C-O-C asymmetric stretching in ester groups. A calculation of the loss in absorbance intensity for these peaks is displayed in Table 3.

In the case of PUD/PET (Figure 7a), the ATR spectra demonstrated a reduction of the absorbance intensity in correspondence of the aforesaid characteristic peaks. This result could be intended as an indication of the harmful effect of UV radiation on the applied PUR to the textiles surface, according to the work of Bhargava et al. [20]. These authors reported that the decrease in the height of these peaks suggested a chain scission mechanism in the PUR binder, due to the aggressive UV exposure, that induced the photo-oxidation mechanism: the bonds in the polyurethane that tend to absorb more energy at smaller wavelengths (such as UV) were directly broken.

Unfortunately, the presence of the organic absorber did not appear to be satisfactory in protecting the PUR polymer from the deteriorating action of UV. In fact also in this case, a diminishing absorbance was always recognized (Figure 7b), for the highlighted suggested peaks, even if this reduction was lower compared to the PUD/PET samples (see Table 3). Yet, in the presence of crosslinking agent, combined with the organic absorber in the dispersion, a similar trend in the absorbance intensity could be detectable for all the tested wavenumber range, by demonstrating a lesser UV aggression towards the chemical bonds [21].

## 4. Discussion and Conclusions

In this work, the final characteristics, in terms of puncture strength and water repellency, of commercial polyester-based technical woven fabrics, impregnated with polyurethane waterborne dispersion, have been studied before and after UV weathering.

The experimental results proved that the fabric impregnation with polyurethane significantly affected the perforating behaviour of the treated materials: the breaking load was increased while the deformation remained almost unaffected. Thus, the presence of the polymer in the textile weaving contributed to greater bonds among the filaments of the pristine fabric, involving more fibers in the sharing of the load. In this way, during the perforation test, a stronger resistance opposed the passage of the penetrator object and a higher force was required for the breakage of the tested textile portion. This consideration was analogously verified in another previous work [22]. For these systems (PUD/PET samples), the water repellency was also sufficiently increased with respect to the measured value for the neat PET. This outcome could be considered a consequence of the distribution of the PUR polymer between the interstices of the weft and warp, and among the constituent filaments of the fabric construction, as verified by SEM micrographs. In this manner, the PUR has produced a thin and transparent, but protective, polymeric film within the textile weaving spaces that limited the passage of water. Nevertheless, the positive benefits coming from the polyurethane treatment on the puncturing strength of the prepared materials were completely lost after the UV aging, probably due to a chemical deterioration of the molecular bonds following UV attack (as attested through ATR analysis). In general, in a composite system, in order to achieve a good migration of stress between the matrix and the fibers, a strong interaction is necessary, such as covalent bonds, and/or secondary forces like van der Waals or hydrogen bonding [23]. Thus, the action of the UV irradiation could also negatively affect the interfacial adhesion between the adopted polymer and the fabric surface, by limiting the adherence and preventing the load transfer. Consequently, in order to preserve over time the obtained characteristics in the developed textiles, other different components were introduced in the aqueous impregnating dispersions in various concentrations: a crosslinking agent and UV light stabilizers (ZnO nanoparticles, Hals, HPT).

The presence of crosslinker in the polyurethane determined a strong reduction of the displacement, evaluated during puncture strength, without a corresponding increase of the breaking load. This had meant a superior rigidity, achieved in the final textile structure, probably for the increasing interactions between the molecular chains (i.e., crosslinking) of the PUR polymer that determined a covalently bonded network and a restriction of the stretching of flexible soft segments through the rigid hard segments [24]. The formation of more urethane linkages in the PUR matrix by adding the crosslinker was just confirmed in our previous similar work, based on the effect of silica nanoparticles/polyurethane waterborne on the perforating features (in terms of spike and blade puncturing strength, and cutting resistance) of impregnated polypropylene-based fabric. In this case, it was established that the crosslinking agent could enhance the strength of the PUR, by analyzing not only the higher blade puncturing and cutting strength in formulations containing the crosslinker agent, but in particular by ATR measurements. In fact, by comparing the IR spectra of impregnated samples with or without crosslinker an increase in the absorbance at 1732 cm^−1^ and of its shoulder at 1690 cm^−1^, the absence of isocyanate (NCO) band at 2270 cm^−1^ and an increase in the urethane characteristic peak (NHC) at 1530 cm^−1^ was verified. All these considerations were interpreted as higher interactions in the PUR macromolecules [22].

Yet, under these conditions, being the puncture mechanism dominated by friction between the yarns and within yarns, rather than by the fiber resistance [25] the more rigid textile weaving hindered the yarns movement during the test, by causing a lesser energy spent in friction. This phenomenon, balancing the greater resistance of the polymer, could lead to maintain the breaking strength almost equal to that of the PUD/PET sample. Anyway, from the experimental data recorded after UV irradiation, the presence of crosslinker continued to be inappropriate in the protection of the textile puncturing strength against the UV attack.

Also, for the dispersions incorporating the UV light stabilizer, even if a lower loss in perforating features (−28%) was determined, compared to the PUD/PET systems (−48.5%), attributed to the UV weathering; no interesting and significant results could be highlighted since the related puncture strength after UV irradiation persisted to be inferior, or no more than similar (in the case of UV organic absorber) compared to that evaluated for the aged PET.

The optimal solution was identified in the impregnating formulations containing both the crosslinker and the UV light stabilizer (in particular for PUD/10Cr/7HPT/PET systems) for which the perforating strength and water repellency were measured higher compared to the neat textile (PET) after UV accelerating aging. In this case, also the ATR spectroscopy confirmed a negligible absorbance loss recorded for the corresponding aged samples. This result could be better explained, taking into account the basic chemical reactions between isocyanate and different reactants described by Chattopadhyay et al. [26], and the possible presence of hydroxyl groups in the chemical structure of the HPT UV absorbers. In fact, as reported in the study of Schaller et al. [27], the crosslinking agent (isocyanate-based) could bind the stabilizer (HPT) in the PUR polymer chain and minimize the problem of its migration to the substrate surface, resulting into a higher durability. Thus, the combination of the two components (Cr together with HPT) could not only have a contribution in preserving the chemical bonds of the polyurethane structure, but also could play a role in protecting the overall performance of the impregnating polymer by preserving also the interfacial adhesion with the textile yarns, mainly responsible of the mechanical load transmission.

**Figure 8:**
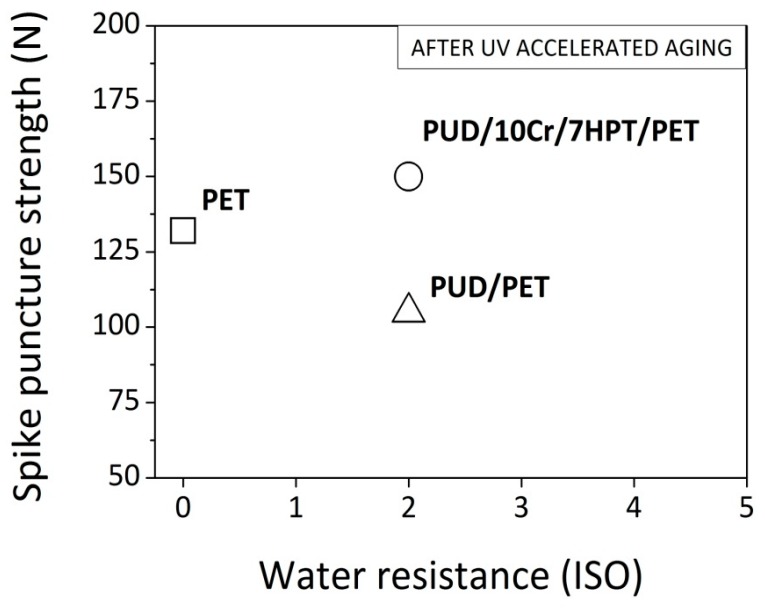
Puncture strength vs water resistance for the main analyzed textiles.

In Figure 8, a conclusive comparison among the main significant textiles (PET, PUD/PET and PUD/10Cr/7HPT/PET) is reported in terms of puncture strength vs water resistance, evaluated after the UV exposure. From the figure, it can be summarized that the characteristics of impregnated textiles (PUD/PET) were superior, in term of water resistance, but inferior, in term of perforation behavior, with respect to the original fabric (PET). On the other side, by implementing the WPUD dispersion with the crosslinker and UV organic absorber, an increase of both features could be obtained.

Finally, the appearance of the developed textiles (PUD/10Cr/7HPT/PET) did not undergo particular changes with respect to the original aspect (PET), as shown in the optical micrographs. In conclusion, waterborne polyurethane dispersions, including an UV light stabilizer and a crosslinking agent, can be considered an effective answer for protecting and improving the performances of commercial or technical fabrics without modifying the initial appearance.

## Figures and Tables

**Table 1:** Components of the prepared impregnating dispersions for a square piece of fabric (sized 20 × 20 cm^2^).

	Mixing Components				
	WPUD	Cr	ZnO	Hals	HPT
PUD	13 g (100%)	/	/	/	/
PUD/10Cr	13 g (100%)	0.78 g (10%) ^1^	/	/	/
PUD/1ZnO	13 g (100%)	/	0.08 g (1%) ^1^	/	/
PUD/4ZnO	13 g (100%)	/	0.32 g (4%) ^1^	/	/
PUD/7ZnO	13 g (100%)	/	0.55 g (7%) ^1^	/	/
PUD/1Hals	13 g (100%)	/		0.15 g ^2^ (1%) ^1^	/
PUD/4Hals	13 g (100%)	/	/	0.62 g ^2^ (4%) ^1^	/
PUD/7Hals	13 g (100%)	/	/	1.06 g ^2^ (7%) ^1^	/
PUD/1HPT	13 g (100%)	/	/	/	0.39 g ^2^ (1%) ^1^
PUD/4HPT	13 g (100%)	/	/	/	1.60 g ^2^ (4%) ^1^
PUD/7HPT	13 g (100%)	/	/	/	2.75 g ^2^ (7%) ^1^
PUD/10Cr/7ZnO	13 g (100%)	0.78 g (10%) ^1^	0.55 g (7%) ^1^	/	/
PUD/10Cr/7Hals	13 g (100%)	0.78 g (10%) ^1^	/	1.06 g ^2^ (7%) ^1^	/
PUD/10Cr/7HPT	13 g (100%)	0.78 g (10%) ^1^	/	/	2.75 g ^2^ (7%) ^1^

^1^ Percentages in table have been calculated with respect to the solid content of WPUD. ^2^ Mass of the aqueous dispersion of UV light stabilizer (HOSTAVIN 3070 DISP or Tinuvin 400) containing the required percentage of active component.

**Table 2:** Load and displacement values, measured during the puncture test, for the prepared samples before and after UV accelerated aging.

	BEFORE UV		AFTER UV	
	Load (N)	Displacement (mm)	Load (N)	Displacement (mm)
(a) Polyurethane impregnation				
PET	160 ± 23	29 ± 8	138 ± 15	19 ± 4
PUD/PET	204 ± 26	26 ± 5	105 ± 10	22 ± 4
(b) Addition of the crosslinker into WPUD				
PUD/10Cr/PET	192 ± 15	19 ± 4	115 ± 14	17 ± 4
(c) Addition of ZnO into WPUD				
PUD/1ZnO/PET	187 ± 18	22 ± 3	107 ± 13	19 ± 4
PUD/4ZnO/PET	203 ± 23	23 ± 7	118 ± 17	17 ± 3
PUD/7ZnO/PET	212 ± 12	24 ± 6	116 ± 10	19 ± 3
(d) Addition of HALS into WPUD				
PUD/1HALS/PET	182 ± 22	22 ± 6	103 ± 16	19 ± 4
PUD/4 HALS/PET	193 ± 23	22 ± 4	100 ± 13	15 ± 3
PUD/7 HALS/PET	176 ± 21	19 ± 4	118 ± 18	17 ± 3
(e) Addition of HPT into WPUD				
PUD/1HPT/PET	187 ± 25	22 ± 4	136 ± 18	17 ± 3
PUD/4HPT/PET	176 ± 24	25 ± 5	126 ± 15	19 ± 4
PUD/7HPT/PET	172 ± 17	23 ± 5	124 ± 15	16 ± 4
(f) Addition of Crosslinker and UV stabilizers into WPUD				
PUD/10Cr/7ZnO/PET	200 ± 27	17 ± 3	142 ± 14	19 ± 3
PUD/10Cr/7HALS/PET	200 ± 17	18 ± 4	142 ± 17	22 ± 3
PUD/10Cr/7HPT/PET	192 ± 15	17 ± 4	149 ± 27	21 ± 4

**Table 3:** Absorbance loss at 2928, 1732, 1530 and 1250 cm^−1^ bands for impregnated textiles after UV treatment.

	Absorbance loss (%)			
	2928 cm^−1^	1732 cm^−1^	1530 cm^−1^	1250 cm^−1^
PUD/PET	32	35	22	24
PUD/7 HPT/PET	28	33	22	16
PUD/10Cr/7HPT/PET	4	10	0	7
